# Late onset of neutral lipid storage disease due to novel *PNPLA2* mutations causing total loss of lipase activity in a patient with myopathy and slight cardiac involvement

**DOI:** 10.1016/j.nmd.2017.01.011

**Published:** 2017-05

**Authors:** Sara Missaglia, Lorenzo Maggi, Marina Mora, Sara Gibertini, Flavia Blasevich, Piergiuseppe Agostoni, Laura Moro, Denise Cassandrini, Filippo Maria Santorelli, Simonetta Gerevini, Daniela Tavian

**Affiliations:** a Laboratory of Cellular Biochemistry and Molecular Biology, CRIBENS, Catholic University of the Sacred Heart, pz Buonarroti 30, Milan 20145, Italy; b Department of Psychology, Catholic University of the Sacred Heart, Largo Gemelli, 1, Milan 20123, Italy; c Neuromuscular Diseases and Neuroimmunology Unit, Fondazione IRCCS Istituto Neurologico “Carlo Besta”, Milan, Italy; d Centro Cardiologico Monzino, IRCCS, Milan, Italy; e Department of Clinical and Community Sciences, University of Milan, Milan, Italy; f Department of Pharmaceutical Sciences, University of Piemonte Orientale “Amedeo-Avogadro”, Novara, Italy; g Molecular Medicine, IRCCS Stella Maris, Pisa, Italy; h Servizio di Neuroradiologia, Ospedale San Raffaele, Milan, Italy

**Keywords:** Neutral lipid storage disease with myopathy, Cardiomyopathy, PNPLA2, Triglyceride lipase, Lipid metabolism, Lipid droplets

## Abstract

Neutral lipid storage disease with myopathy (NLSDM) presents with skeletal muscle myopathy and severe dilated cardiomyopathy in nearly 40% of cases. NLSDM is caused by mutations in the *PNPLA2* gene, which encodes the adipose triglyceride lipase (ATGL). Here we report clinical and genetic findings of a patient carrying two novel *PNPLA2* mutations (c.696+4A>G and c.553_565delGTCCCCCTTCTCG). She presented at age 39 with right upper limb abduction weakness slowly progressing over the years with asymmetric involvement of proximal upper and lower limb muscles. Cardiological evaluation through ECG and heart echo scan was normal until the age 53, when mild left ventricular diastolic dysfunction was detected. Molecular analysis revealed that only one type of *PNPLA2* transcript, with exon 5 skipping, was expressed in patient cells. Such aberrant mRNA causes the production of a shorter ATGL protein, lacking part of the catalytic domain. This is an intriguing case, displaying severe *PNPLA2* mutations with clinical presentation characterized by slight cardiac impairment and full expression of severe asymmetric myopathy.

## Highlights

• Two novel mutations in *PNPLA2* gene have been identified in a neutral lipid storage disease with myopathy (NLSDM) female patient.
• The mutations are located in exon 5 of *PNPLA2* and abrogate lipase function.
• The patient showed late onset skeletal muscle myopathy and mild cardiac impairment.
• Clinical cardiac phenotype is milder in NLSDM female patients, beyond genetics.

## 1 Introduction

Neutral lipid storage disease with myopathy (NLSDM; MIM 610717) is an autosomal recessive disorder characterized by abnormal accumulation of triacylglycerols (TAGs) in cytoplasmic lipid droplets (LDs) in most tissues, including muscle, heart, liver and peripheral blood. A rapid laboratory diagnosis of NLSDM can be easily performed through the detection of lipid vacuoles in peripheral blood leucocytes, also known as Jordans' anomaly [1], [2]. To our best knowledge, forty-six NLSDM patients have been clinically and genetically reported [3], [4], [5], [6], [7], [8], [9], [10], [11], [12], [13]. Clinical symptoms of NLSDM are characterized by progressive myopathy, cardiomyopathy, hepatomegaly, diabetes, chronic pancreatitis, short stature and by high serum creatine kinase levels [14]. The degree of clinical manifestations appears highly variable: from minimal symptoms to a more severe condition, causing physical disability and premature death due to dilated cardiomyopathy. However, since NLSDM is a rare metabolic condition, the pathophysiology of the disease is largely unclear and phenotype–genotype correlations remain incomplete [15]. NLSDM is caused by mutations in *PNPLA2* coding for the adipose triglyceride lipase (ATGL), a member of the patatin-like phospholipase domain-containing proteins [3]. This lipase is a lipid droplet-associated protein that catalyses the first step in the hydrolysis of TAGs, stored within LDs [16]. The human ATGL protein consists of 504 amino acids comprising the patatin domain (amino acids 10–178) with catalytic residues S47 and D166, at the N-terminus, and a hydrophobic lipid binding domain at position 315–360 towards the C-terminus [3], [16].Thirty-five *PNPLA2* mutations variably affecting protein function or production have been identified so far in NLSDM patients. Many mutations are expected to generate either null alleles or truncated ATGL proteins with the catalytic domain partially lost, all resulting in dramatic impairment of LD metabolism. The outcome in most patients carrying these mutations has been reported as severe. On the contrary, recent studies showed that missense mutations, resulting in an ATGL protein with residual lipolytic activity, may be associated with slowly progressing myopathy and sparing of myocardial muscle [9], [12], [17]. Here we describe clinical and genetic findings in a woman harbouring two novel mutations in *PNPLA2.* Although these mutations completely abolish lipase activity, our patient showed slowly progressive skeletal muscle weakness with late presentation, in association with mild cardiac impairment.

## 2 Case presentation

**Fig. 1:**
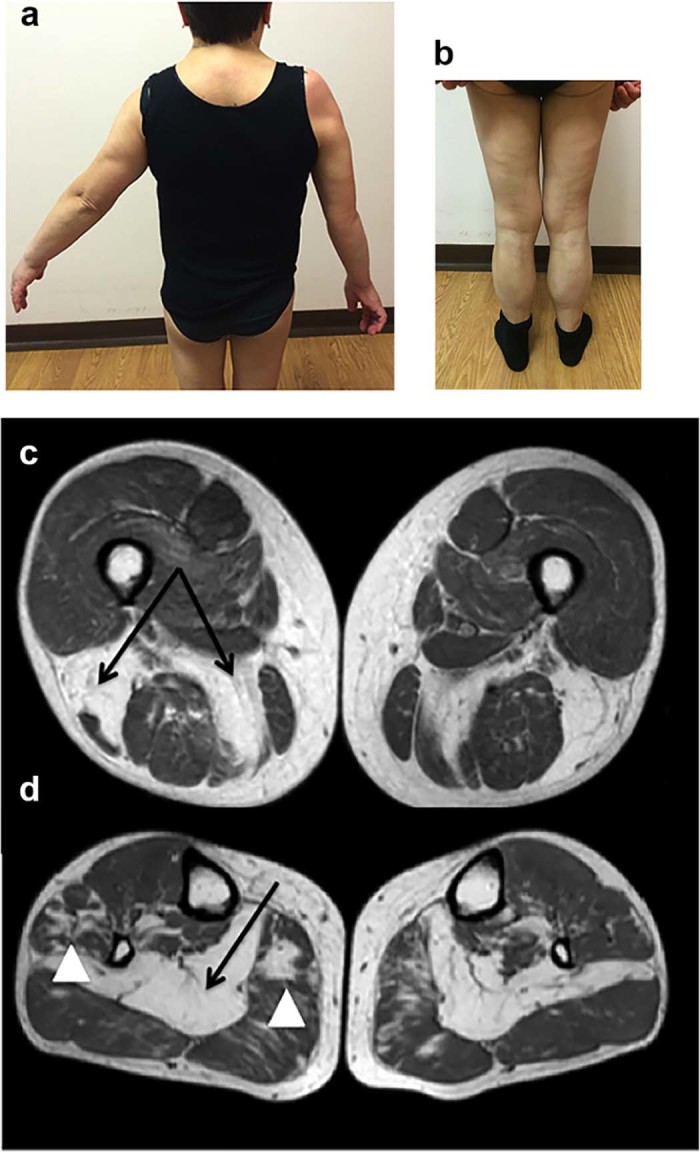
NLSDM patient's clinical and imaging features. (a) Upper limb abduction weakness, predominant on the right side and (b) calves hypertrophy. Thigh (c) and leg (d) muscle MRI: marked T1w changes with almost complete fatty infiltration of biceps femoris and semimembranosus (c) and soleus (d); lower severity involvement has been observed in medial gastrocnemius and peroneal longus (arrowheads) (d).

The proband is a 54-year-old woman, presenting at age 39 with right upper limb proximal weakness, slowly progressing over the years. Her previous medical history had been unremarkable. At age 47 she noticed muscle weakness in lower limbs. Creatine kinase was 579 U/L (normal range < 190 U/L). Urine organic acids, plasma carnitine and acyl-carnitine profiles were normal. Electromyography performed elsewhere showed a mixed pattern with predominant neurogenic signs and fibrillations in upper limb muscles; nerve conduction studies were normal. The patient, when first admitted in our outpatient clinic at age 49, reported moderate disability due to upper limb muscle weakness. No bulbar symptoms, muscle cramps or pain was reported. Neurological examination showed marked right upper limb weakness with abduction limited to 30 degrees and flexion to 45 degrees, whereas only mild weakness against resistance was observed on the left side. In addition, severe elbow flexion and moderate elbow and finger extension weakness were noticed on the right side. No axial or lower limb weakness was found (Fig. 1a and b). Mild hypertrophy of calves was observed. Tendon retractions or scapular winging was absent. Cranial nerves were normal. Pyramidal or cerebellar signs were absent; deep tendon reflexes were reduced in lower limbs, while triceps and biceps reflexes were inelicitable; superficial and deep sensibility were normal. Muscle MRI, performed at age 49, showed predominant posterior thigh and leg compartment involvement (Fig. 1c and d), as already reported [7].

**Fig. 2:**
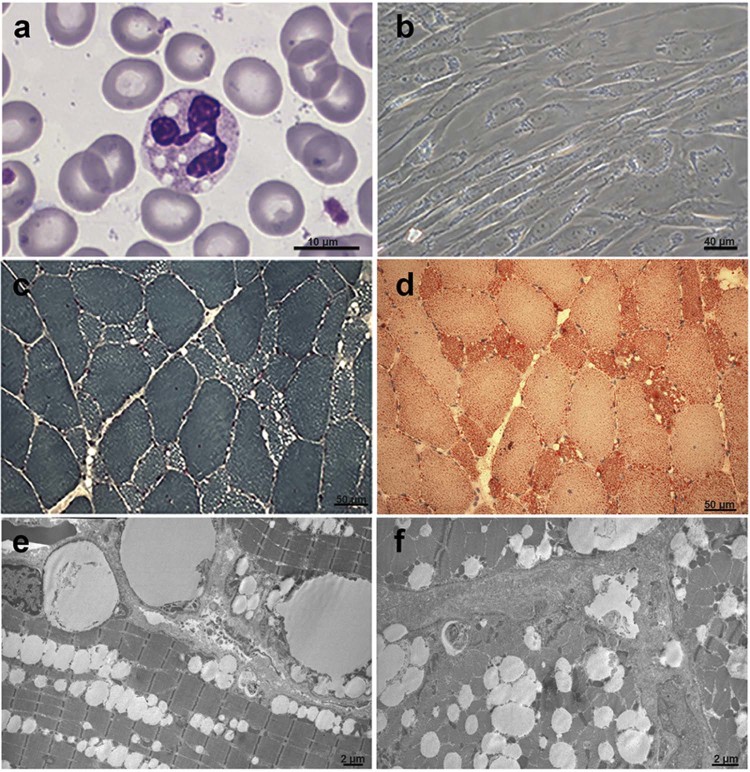
Histochemical characterization of NLSDM patient. (a) Detection of Jordans' bodies in granulocytes, stained with May-Grünland Giemsa (MGG). (b) Phase contrast image of cultured fibroblasts from the patient reveals increase of lipid droplet storage inside the cells. Consecutive cryosections of the patient muscle biopsy, stained with Gomori trichrome (c) and Oil-Red-O (d), show microvacuoles and abnormal accumulation of lipids. (e,f) Electron microscopy reveals massive line-up of lipid droplets without signs of mitochondrial alteration.

A quadriceps muscle biopsy, performed at the same age, revealed myogenic features with vacuoles mainly distributed in hypotrophic type I fibres; staining with Oil Red O showed lipid accumulation (Fig. 2d). No degeneration or regeneration was observed (Fig. 2c). Electron microscopy confirmed the excessive accumulation of lipid droplets without signs of mitochondrial alteration (Fig. 2e and f). Jordans' anomaly was found in the patient leucocytes at age 51 (Fig. 2a). Cultured skin fibroblasts, obtained from a patient dermal biopsy, also revealed an abnormal accumulation of neutral lipids into LDs (Fig. 2b).

The neurological follow-up showed moderate worsening of the condition and development of moderate muscle weakness of lower limbs, mainly in proximal muscles. In addition, neck flexor muscles were also impaired. The patient remained fully ambulant during the follow-up period.

Cardiological evaluation through ECG and heart echo scan were normal until the age 53, when mild left ventricular diastolic dysfunction was detected, without any progression over the following 12 months. At age 54, heart MRI and standard spirometry were normal while a cardiopulmonary exercise test showed an exercise limitation (peak VO_2_ = 66% predicted value). All together the cardiopulmonary exercise test data were suggestive of peripheral muscle deconditioning with normal cardiac function.

**Fig. 3:**
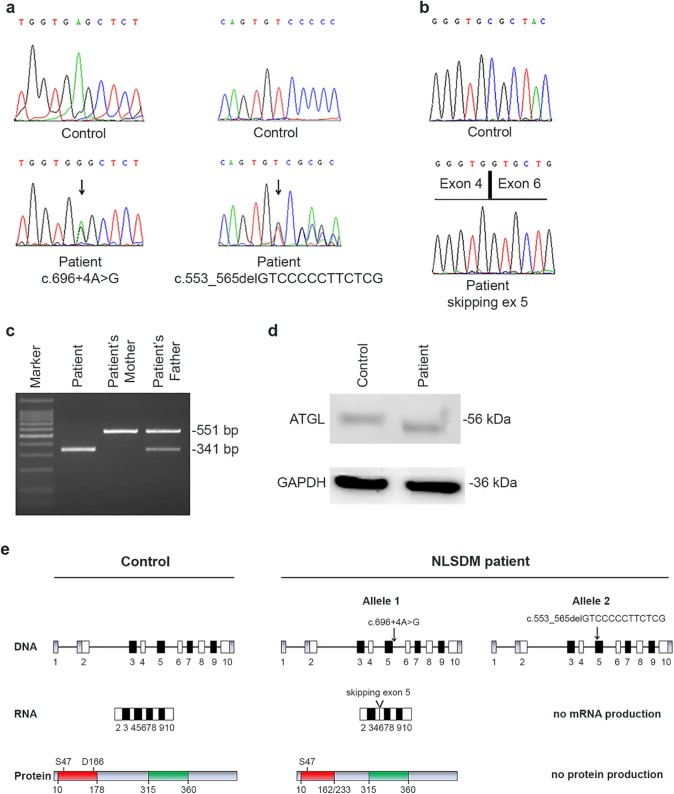
Molecular characterization of *PNPLA2* mutations. (a) Electropherograms of *PNPLA2* exon 5 showing a splice-site mutation (c.696+4A>G) in the first allele and a deletion of 13 bp (c.553_565delGTCCCCCTTCTCG) in the second allele of the patient. (b) Electropherogram of RT-PCR products reveals the skipping of exon 5 in the patient. (c) RT-PCR performed with primers encompassing exons 3, 4, 5 and 6 shows absence of wild-type product (551 bp) in the patient and the presence of a single product of 341 bp, resulting from c.696+4A>G mutation. Patient's mother presents a single wild-type allele and father carries the c.696+4A>G mutation in heterozygous status. (d) Western blot analysis of fibroblasts' extracts shows the wild-type product (56 kDa) in control cells and the mutant protein (48 kDa) in NLSDM fibroblasts. GAPDH has been used as loading control for protein normalization (36 kDa). (e) Schematic representation of the *PNPLA2* gene, mRNA and protein in the control subject and in the Italian patient. The diagram of normal PNPLA2 is reported on the left. ATGL protein wild type contains a patatin domain (red) with the catalytic dyad (S47 and D166) and a hydrophobic domain (green). *PNPLA2* mutations identified in the NLSDM subject determine: in the first allele (c.696+4A>G) the skipping of exon 5 and the production of a mutant protein lacking part of catalytic site (D166 amino acid); in the second allele (c.553_565delGTCCCCCTTCTCG) the lack of mRNA expression and protein production.

Family history was negative for neuromuscular disorders. Parents were not consanguineous. In our patient, molecular analysis of *PNPLA2* detected the two following novel heterozygous mutations: c.696+4A>G (allele 1) and c.553_565delGTCCCCCTTCTCG (allele 2) (Fig. 3a). The first mutation, inherited from the father, is localized in intron 5 and predicts in frame skipping of exon 5. The aberrant mRNA loses part of the sequence coding for the catalytic site of ATGL protein (Fig. 3b and c). The deletion extends from Arg163 to Leu232, including the Asp166 residue, which is part of the catalytic dyad (Fig. 3e). Hence, the c.696+4A>G mutation, disrupting the ATGL catalytic site, causes total loss of its enzymatic function, as previously shown by functional studies [9], [12], [17]. The c.553_565delGTCCCCCTTCTCG mutation is localized inside exon 5; extensive RT-PCR analysis showed no *PNPLA2* mRNA production from this mutant allele (Fig. 3c). This variant was carried also by the 81-year-old mother, who, at age 77, was normal on neurological examination and had a normal muscle biopsy. Both *PNPLA2* mutations were not observed in >200 control alleles and were submitted to GenBank (Accession numbers: KU139128 for c.696+4A>G and KU139129 for c.553_565delGTCCCCCTTCTCG).

To verify whether patient allele 1, showing the in frame skipping of exon 5, was expressed into patient cells, we performed western blotting analysis of ATGL using total protein extracts from patient fibroblasts. As shown in Fig. 3d, a mutated ATGL protein with lower molecular weight was detected in patient fibroblasts in comparison with control fibroblasts.

Informed consent was obtained from the study participants. Patient investigations were conducted in accordance with protocols approved by the institutional review boards of the Carlo Besta Neurological Institute and the Catholic University of the Sacred Heart.

## 3 Discussion

The main clinical feature of NLSDM is skeletal muscle myopathy, which is present in 100% of patients. Muscle weakness usually presents in early adult life, between 20 and 30 years. A later onset of the muscle phenotype has been observed in our patient, as well as in some previously reported cases [7], [8], [9], [12], [18]. In these patients, mainly *PNPLA2* missense mutations, which partially save lipase activity, have been identified. On the contrary, in our patient with typical muscle phenotype characterized by predominant proximal upper limb muscle weakness, two severe mutations that completely abrogate protein function have been detected. The first mutation causes the skipping of exon 5 and the production of a mutated protein that loses part of the catalytic site, thus abrogating ATGL lipase activity; the second mutation determines complete lack of mRNA expression and protein production (Fig. 3e). A homozygous *PNPLA2* mutation affecting the invariant G of the donor splice-site of intron 5 (c.696+1G>C) has previously been described in a Japanese male patient [19]. This mutation caused the production of two aberrant mRNAs: one retaining 93 bp of intron 5 and resulting in a new reading frame shift and a stop at position 162 (p.Val233LeufsX162); the other consisting of a *PNPLA2* sequence lacking 210 bp due to in frame skipping of exon 5 (p.Arg163_Leu232del), exactly as the allele 1 of our female patient (with c.696+4A>G mutation). Despite the molecular similarity, some important clinical differences emerged between the Japanese and the Italian patients, concerning, in particular, their cardiac involvement. These differences might be due to homozygous versus heterozygous condition or to modifier genes and epigenetic factors possibly involved in such variable phenotypic expression. In the Japanese patient muscle weakness presented earlier than in our patient and was associated with severe heart involvement presenting at age 33 and requiring heart transplantation. On the contrary, our female patient showed only slight cardiac involvement at age 53.

Although the presence of *PNPLA2* severe mutations is similar in men and women, cardiac damage was reported in almost 20% of NLSDM female patients (4 out of 20) and in 55% of male patients (15 out of 27) [13], [20]. Indeed, considering as severe the mutations (identified so far in NLSDM patients) that cause lack of ATGL protein production or expression of truncated proteins with catalytic site only partially conserved, we note that they represent 25% of total *PNPLA2* mutations in female patients and 29% in male patients. The latter observation suggests that gender modulates clinical cardiac phenotype in NLSDM, also beyond the severity of mutations in *PNPLA2*. To this regard, it is known that oestrogens regulate the expression of peroxisome proliferator-activated receptor (PPARα,β/δ,γ) family members. PPARs control mitochondrial metabolism and are mainly involved in fatty acid and glucose utilization in heart [21], [22]. Very recently, Higashi et al. reported a distinct cardiac phenotype in two NLSDM siblings carrying the same homozygous *PNPLA2* mutation; according to the hypothesis that there is a gender difference on the phenotypic clinical expression in NLSDM, the male died at age of 31 of heart failure, while his sister was still alive (44 years), although presenting hypertrophic cardiomyopathy [20]. Certainly, additional larger clinical studies are warranted to elucidate whether female gender possibly plays a protective role in NLSDM. NLSDM is an ultra-rare disease, its pathophysiology is largely unclear, phenotype–genotype correlations are incomplete, and a cure is still lacking. To this regard, an international registry for NLSDs, recently established (www.tgcv.org/r/home.htlm), may help to collect worldwide clinical and genetic data and develop common therapeutic protocols.

In conclusion, we describe a 54-year-old NLSDM female patient showing late onset myopathy in association with slight cardiac involvement, although the identified novel mutations completely abrogate *PNPLA2* protein function. Our data expand the allelic spectrum of *PNPLA2* mutations, providing further evidence for genetic and clinical NLSDM heterogeneity.

## Funding

This work was supported by grant GGP14066 from Telethon Foundation.
